# Combination of Focused Assessment With Sonography for Trauma (FAST) Scan and Detection of Hematuria to Exclude Intra-abdominal Injuries Following Blunt Abdominal Trauma

**DOI:** 10.7759/cureus.34736

**Published:** 2023-02-07

**Authors:** Mohammad A Abd-erRazik, Mohamed A Abdel Hamid, Mohamed El-Shinawi, Jon M Hirshon, Hazem M El-Hariri, Maged El-Setouhy

**Affiliations:** 1 Department of General Surgery, Ain Shams University, Cairo, EGY; 2 Department of Surgery, Galala University, Suez, EGY; 3 Department of Emergency Medicine, University of Maryland School of Medicine, Baltimore, USA; 4 Department of Emergency Medicine/Community Medicine, University of Maryland School of Medicine, Baltimore, USA; 5 Department of Community Medicine, National Research Centre, Cairo, EGY; 6 Department of Family and Community Medicine, Jazan University, Jazan, SAU; 7 Department of Community, Environmental, and Occupational Medicine, Ain Shams University, Cairo, EGY

**Keywords:** focused assessment with sonography for trauma, blunt abdominal injury, blunt abdominal trauma, intra-abdominal injuries, hematuria, fast scan

## Abstract

Background

Blunt abdominal trauma (BAT) is the most common pattern of abdominal traumas. It may be associated with intra-abdominal injuries (IAIs). Exploratory laparotomies are only needed in a minority of patients after BAT.

Methodology

All BAT patients who presented to the El Demerdash Hospital of Ain Shams University, Cairo, Egypt during the study period were traced. Parameters including demographic data, focused assessment with sonography for trauma (FAST) scan, CT scan results, and hematuria were collected. The cohort was divided according to the CT scan results into two groups: patients with IAIs and patients without IAIs.

Results

Males represented 78.2% of the patients, and the mean age of the recruited patients was 32.1 ± 18 years. Road traffic accidents represented the main cause of trauma (58%)*. *Patients with IAIs detected by CT scan represented 1.62%, and hematuria was detected in 88.9% of them. The specificity of FAST was 97.1%, and that of hematuria was 84.1%, and for the combination of both tests, the specificity was 99.3%.

Conclusion

IAIs after BAT can usually be excluded if both FAST and hematuria are negative, provided that the patient is stable.

## Introduction

Unintentional injuries are the leading cause of death in age groups from one to 45 years, with road traffic accidents being the primary source of these injuries [1,2]. Abdominal injuries constitute 15% to 20% of trauma-related cases, resulting in a mortality rate of 10% to 30% among those affected [3]. Blunt abdominal trauma (BAT) accounts for over 80% of all abdominal traumas, with associated intra-abdominal injuries (IAIs) either retroperitoneal or intraperitoneal, with the latter further divided into solid and hollow organ injuries [4]. Solid organ injuries are more frequent, with liver injuries being the most commonly injured organ in BAT [5]. Exploratory laparotomies are needed in only 4% of cases [4], so excluding IAIs is the main basis for deciding on the non-operative management of BAT.

Physicians depend on abdominal signs and symptoms, or lack of them, as triage criteria for further workup [6]. However, these cannot be considered reliable indicators of the presence or absence of IAIs. So several objective assessments are often employed to aid the diagnosis, such as focused assessment with sonography for trauma (FAST), extended focused assessment with sonography for trauma (eFAST), CT scan, diagnostic peritoneal lavage (DPL), or even diagnostic exploratory laparotomy and laparoscopy [6,7].

FAST is a commonly available modality for the assessment of IAIs in trauma patients. It is a non-invasive, relatively cheap method, with no risk of radiation. However, it is operator-dependent. The FAST sensitivity is 90% (75-100%) and the specificity is 95% (88-100%) [8,9]. A positive FAST with hypotension in BAT patients is an indication of laparotomy. On the other hand, a negative or equivocal FAST, especially with hypotension, cannot safely exclude IAIs [10,11]. The CT scan is an important tool in the diagnosis of IAIs, but it has its risks and drawbacks. The main risk is exposure to ionizing radiation. Moreover, due to the nature of the study, the patient needs to be stable on the machine and to hold his breath when he is ordered to do so; this makes the study more challenging for agitated, confused, and pediatric patients.

Hematuria is the presence of blood in the urine. The word has a Greek origin from two words *haima *(blood) and *ouron* (urine) [12]. The definition of gross or macroscopic hematuria is straightforward, which is the hematuria noticed clinically by the naked eye. While microscopic hematuria is more challenging in its definition, as it may be defined as follows: >2 red blood cells per high power field (RBC/HPF) in two microscopic urine analyses [13], ≥ 3 RBC/HPF [14], >3 RBC/HPF [15], five RBC/HPF, or 20 RBC/µL = +1 dipstick test [16].

Hematuria used to be a proposed indicator for genitourinary injuries (GUIs) and IAIs [17,18]. It is commonly accepted that gross hematuria is a marker for both GUI and IAI, yet, the significance of microscopic hematuria remains questionable [19,20].

The purpose of this study was to assess the combined use of FAST and detection of hematuria in the assessment of BAT patients, and whether this combination could safely exclude the presence of IAIs or not.

## Materials and methods

The study was a concurrent cohort study, including all the BAT patients who presented to the emergency room of El Demerdash Hospital. It is a quaternary hospital, which is the surgical hospital of Ain Shams University Hospitals, Cairo, Egypt. On average, it has more than 7,000 annual emergency room visits for diagnoses related to trauma [21]. It has around 15,500 emergency admissions yearly, more than 2,500 of them are trauma patients, and about 800 patients are discharged within 24 hours after being stabilized and investigated. The study period was between November 2018 and October 2019. Included patients were those who presented within 24 hours after the trauma and with a Glasgow Coma Scale score greater than 8 to ensure a reliable examination, and were assessed by FAST. Exclusion criteria were as follows: BAT patients discharged without a CT scan, patients with a prehospital urinary catheterization, patients with a known history of bleeding disorders, patients with liver cirrhosis or a known history of ascites, and those with urinary diversion.

All patients underwent the usual pathway of trauma patients adopted by the hospital, which included history taking, examination (primary and secondary surveys), trauma laboratory studies, and radiologic studies, which included a FAST and an abdominopelvic CT scan. The FAST was performed for all patients by a well-trained radiology specialist registrar. The presence of hematuria was tested in the urine samples using a dipstick test (Medi-Test Combi 11, MACHEREY-NAGEL GmbH & Co. KG, Düren, Germany).

**Figure 1:**
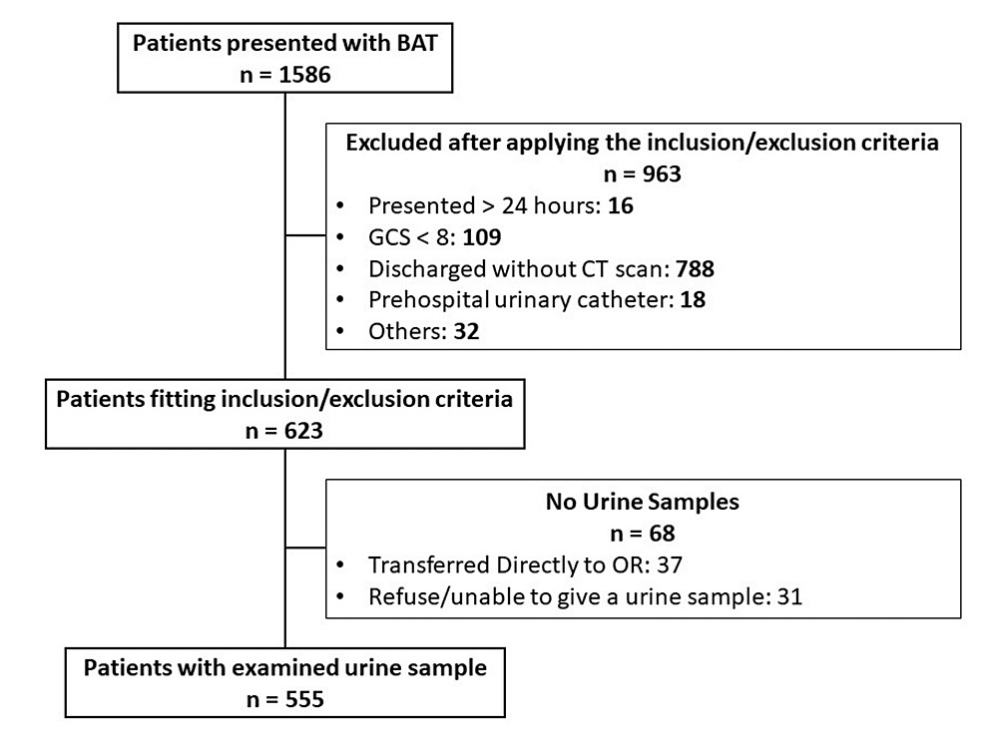
Workflow of the patients BAT: blunt abdominal trauma; GSC: Glasgow Coma Scale; CT: computed tomography; OR: operating room.

Patients were subdivided into two groups according to the findings of the CT scan. The workflow of the patients is shown in Figure 1.

Ethical approvals

The study was approved by both the Research Ethics Committee (REC), General Surgery Department, Ain Shams University (IRB: 00006379), and the University of Maryland, Baltimore (UMB) Institutional Review Board (IRB), and it followed the tenets of the Declaration of Helsinki.

Operational definitions

“Road traffic crashes (RTC)” are all accidents related to moving vehicles, the patient may be the drivers, vehicle passengers, or pedestrians. “Falling from a height” is falling from one or more story heights, i.e., more than three meters. “Falling” is falling from less than one story height, like slips, stumbles, or falling down stairs.

Statistical analysis

The collected data were coded, tabulated, and statistically analyzed using IBM SPSS Statistics software version 22.0 (IBM Corp., Armonk, NY).

The descriptive statistics were done for the quantitative data as mean ± SD, and as number and percentage for the qualitative data. The inferential analyses were done for the quantitative variables using the independent t-test; while for the qualitative data, the inferential analyses for the independent variables were done using the chi-square test for differences between proportions and the Fisher’s exact test for the variables with small expected numbers. A p-value less than 0.05 was considered to be statistically significant, otherwise, it is non-significant.

Availability of data and materials

The datasets used and analyzed during the current study are available from the corresponding author upon reasonable request.

## Results

Data were collected from 1,586 patients presented with BAT. Only 555 patients were enrolled after meeting the inclusion/exclusion criteria, of whom, 434 were males and 121 were females. The mean age was 32.1 ± 18 years. The demographic data and the comorbidities are demonstrated in Table 1.

**Table 1:** Demographic characteristics and comorbidities DM: diabetes mellitus; CLD: chronic liver disease; IHD: ischemic heart disease.

Variables		All cases (n = 555), n (%)
Age (years), mean ± SD		32.1 ± 18.0
Gender	Males	434 (78.2%)
	Females	121 (21.8%)
Comorbidity free		459 (82.27%)
Hypertension		43 (7.7%)
DM		42 (7.6%)
CLD		14 (2.5%)
IHD		6 (1.1%)
Epilepsy		4 (0.7%)

**Figure 2:**
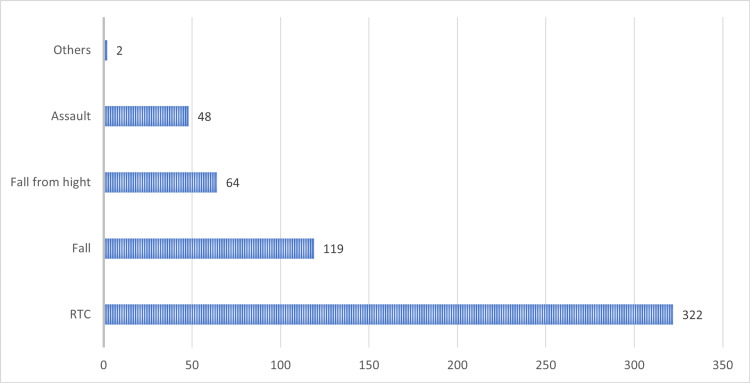
Mode of trauma among the patients RTC: road traffic crashes.

Modes of trauma among the patients are shown in Figure 2. There were no statistically significant differences between the two groups.

The vital data were presented in Table 2. Pulse and systolic and diastolic arterial blood pressures showed a statistically significant difference between the two groups. The urine dipstick findings are reported in Table 3. Hematuria was more prevalent in patients with positive CT (88.9%), and there was a statistically significant difference.

**Table 2:** Vital data Statistically significant values are in bold. bpm: beats per minute; BP: blood pressure; ^ independent t-test; * significant (<0.05).

Variables	All cases (N = 555) (mean, SD)	Positive CT (N = 9) (mean, SD)	Free CT (N = 546) (mean, SD)	P-value
Pulse (bpm)	87.9 ± 13.2	99.0 ± 18.5	87.7 ± 13.0	^0.011*
Systolic BP (mmHg)	117.9 ± 17.2	104.8 ± 16.1	118.1 ± 17.2	^0.021*
Diastolic BP (mmHg)	77.0 ± 11.9	63.9 ± 14.4	77.2 ± 11.7	^0.001*
Temperature (°C)	36.89 ± 1.5	36.97 ± 1.5	37.1 ± 0.4	^0.083

**Table 3:** Urine dipsticks results Statistically significant values are in bold. Sp gravity: specific gravity; ^ independent t-test; § Fisher's exact test; * significant (<0.05).

Variables	All cases (N = 555) (n, %)	Positive CT (N=9) (n, %)	Free CT (N=546) (n, %)	P-value
Sp gravity (n ± SD)	1015.0 ± 9.5	1015.0 ± 13.5	1015.0 ± 9.5	^0.999
pH (n ± SD)	5.7 ± 0.8	5.7 ± 0.9	5.7 ± 0.8	^0.972
Urobilinogen	33 (5.9%)	2 (22.2%)	31 (5.7%)	§0.095
Bilirubin	55 (9.9%)	0 (0.0%)	55 (10.1%)	§0.610
Ketones	23 (4.1%)	0 (0.0%)	23 (4.2%)	§0.999
Glucose	121 (21.8%)	1 (11.1%)	120 (22.0%)	§0.691
Proteins	69 (12.4%)	1 (11.1%)	68 (12.5%)	§0.999
Nitro	4 (0.7%)	0 (0.0%)	4 (0.7%)	§0.999
Leukocytes	28 (5.0%)	0 (0.0%)	28 (5.1%)	§0.999
Hematuria	95 (17.1%)	8 (88.9%)	87 (15.9%)	§<0.001*

**Figure 3:**
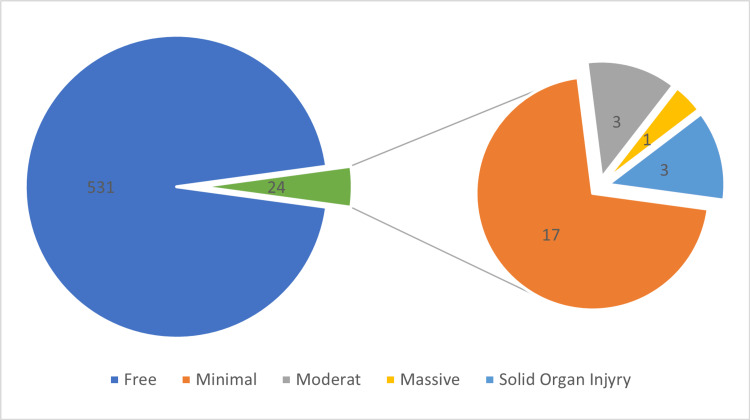
The focused assessment with sonography for trauma (FAST) reports

The results of the FAST scan are shown in Figure 3. Of the patients, 95.7% (n = 531) had a free scan, while only 24 (4.3%) patients had a positive FAST scan. Fluid collections were reported in 21 (3.8%) patients, while an injury of a solid organ was reported in three (0.5%) patients. Out of the nine cases with positive CT scans, eight (88.9%) had positive FAST results. This was a statistically significant difference with a p-value < 0.001.

Table 4 shows the characteristics of FAST and hematuria as tests for spotting IAIs and also shows the effects of combining them both. The FAST scan had high specificity, negative predictive value (NPV), diagnostic accuracy (DA), and diagnostic odd ratio (DOR); such characteristic values were higher than those of hematuria. Both tests had moderate values for other characteristics but were slightly higher with FAST. However, both tests had equal sensitivity.

**Table 4:** Diagnostic characteristics of hematuria and FAST findings in diagnosing intra-abdominal injuries Statistically significant values are in bold. FAST: focused assessment with sonography for trauma; DA: diagnostic accuracy; PPV: positive predictive value; NPV: negative predictive value; LR+: positive likelihood ratio; LR-: negative likelihood ratio; DOR: diagnostic odd ratio.

Characteristics	FAST	Hematuria	Both of them
Sensitivity	88.9% (51.8%–99.7%)	88.9% (51.8%–99.7%)	77.8% (40.0%–97.2%)
Specificity	97.1% (95.3%–98.3%)	84.1% (80.7%–87.0%)	99.6% (98.7%–100.0%)
DA	96.9% (95.1%–98.2%)	84.1% (80.8%–87.1%)	99.3% (98.2%–99.8%)
Youden's index	86.0% (65.4%–106.5%)	73.0% (52.2%–93.7%)	77.4% (50.2%–100.0%)
PPV	33.3% (15.6%–55.3%)	8.4% (3.7%–15.9%)	77.8% (40.0%–97.2%)
NPV	99.8% (99.0%–100.0%)	99.8% (98.8%–100.0%)	99.6% (98.7%–100.0%)
LR+	30.33 (17.76–51.80)	5.58 (4.13–7.54)	212.33 (50.98–884.41)
LR-	0.11 (0.02–0.73)	0.13 (0.02–0.84)	0.22 (0.07–0.76)
DOR	265.00 (31.26–2246.77)	42.21 (5.21–341.73)	952.00 (116.93–7750.70)
Kappa	0.472 (0.262–0.683)	0.128 (0.045–0.211)	0.774 (0.559–0.989)

The combination of both FAST and hematuria increased the specificity, positive predictive value (PPV), DA, positive likelihood ratio (LR+), and DOR, with nearly the same NPV, but it decreased other characteristic values, including sensitivity.

## Discussion

In this study, BAT patients were traced during their workup in El Demerdash Hospital. Males were shown to be more vulnerable to trauma; they represented 78% of BAT patients in this study. Similar figures (73-83%) were reported in the literature [9,11,21-23]; however, slightly smaller figures (58-66%) were also reported in other studies [6,24-26]. The mean age of BAT victims in this study was 32.1 years. This mean was similar to that reported by other studies done in Egypt, which ranged from 32.7-34.2 years old [2,21,27]. The figures we reported in this study may represent a more accurate value, due to the larger sample size.

The most common mode of trauma (58%) was the RTC. It is always reported as the most frequent cause of BAT in both developing [26] and developed [1] countries. The second most common mode of trauma in most publications [9,28], similar to here, is fall (21.4%). Then comes fall from a height, which represents 11.5%, similar to that reported by Samuel et al., who reported an incidence of 12.7% [9]. Some studies do not differentiate between falling and falling from a height [9,28-30]. In our opinion, it is of great importance to distinguish between these two diagnoses during both history taking and data recording. The diagnosis of falling from a height will triage the patient as a patient with a violent mode of trauma with a high possibility of deceleration injuries, major fractures, and multiple internal organs injuries.

The FAST scan is considered to be the most valuable innovation in the assessment of patients with BAT since the introduction of the DPL [31]. It is usually reported as positive or negative. But in this study, because of the hospital settings, the FAST had been done by a well-trained radiology specialist, which is why they tend to report more details; for example, the amount of the present fluid, and also to report any striking findings during the examination like solid organ lacerations. Positive FAST in hemodynamically stable BAT patients does not mandate laparotomy (immediate or delayed) [32].

Hematuria has traditionally been considered a consistent sign of GUI [19]. Studies had suggested that hematuria is a good predictor of IAIs, including GUI, after a BAT in the pediatric age group [18,33]. Others suggested that it can predict IAIs after a BAT in both adults and pediatrics [19,34], or at least can safely exclude IAIs [35]. Some authors reported low sensitivity and specificity of hematuria in the detection of IAIs [36].

Hematuria was detected in this study with a urine dipstick test, which technically is based on the pseudo-peroxidative activity of hemoglobin and myoglobin. This activity catalyzes the oxidation of an indicator, producing a green color. According to the manufacturer, this test can detect as low as 4 erythrocytes/μL (corresponding to approximately 0.012 mg hemoglobin or myoglobin/dL). The exact causes and pathophysiology behind microhematuria associated with IAIs are not clear in the literature. It may be attributed to capillaries disruption in the kidneys resulting from the trauma force [18].

Both FAST and dipstick tests are readily available and relatively cheap options. Each of them alone cannot safely prove or exclude IAIs. This study was testing the effect of combining both of them. On the combination of these tests, the sensitivity decreases, which results in increasing false-positive patients, leading to exhausting the resources to investigate them. On the other hand, this combination significantly increases the specificity, DA, NPV, and DOR. Those specific characteristics will allow excluding IAIs in patients having negative FAST and urine tests, especially if they have normal heart rate and blood pressure. This tool is of value in areas or situations where there is no luxury of fully investigating the patient.

Limitations

The study has several limitations. One limitation is that the strict inclusion/exclusion criteria resulted in the recruitment of only 40% or less of the potential population of the study. Another limitation is that some patients refused or were unable to give urine samples while in the emergency room. Additionally, patients who were immediately transferred to the operating theater based on their vital data or other findings were not included in the study, even though they potentially have a higher incidence of IAIs.

## Conclusions

The use of FAST in combination with hematuria detection has been shown to significantly improve the specificity of detecting IAIs after BAT. Also, the PPV, DA, LR+, and DOR are all improved when these two tests are used together.

When both FAST and hematuria are negative and the patient has normal heart rate and blood pressure, it is highly likely that IAIs can be safely ruled out. However, more prospective and randomized controlled trials with larger cohorts are needed to support and increase the level of evidence for these findings.
